# Capnography waveforms: basic interpretation in neonatal intensive care

**DOI:** 10.3389/fped.2024.1396846

**Published:** 2024-04-04

**Authors:** Emma E. Williams, Theodore Dassios, Christopher Harris, Anne Greenough

**Affiliations:** ^1^ Department of Women and Children’s Health, School of Life Course Sciences, Faculty of Life Sciences and Medicine, King’s College London, London, United Kingdom; ^2^ Neonatal Intensive Care Centre, King’s College Hospital NHS Foundation Trust, London, United Kingdom

**Keywords:** capnography, infant, end-tidal, ventilation, waveform

## Abstract

End-tidal capnography can provide useful clinical information displayed on the ventilator screen or bedside monitor. It is important that clinicians can assess and utilise this information to assist in identifying underlying complications and pulmonary pathology. Sudden change or loss of the CO_2_ waveform can act as a safety measure in alerting clinicians of a dislodged or blocked endotracheal tube, considering the concurrent flow and volume waveforms. Visual pattern recognition by the clinicians of commonly seen waveform traces may act as an adjunct to other modes of ventilatory monitoring techniques. Waveforms traces can aid clinical management, help identify cases of ventilation asynchrony between the infant and the ventilator. We present some common clinical scenarios where tidal capnography can be useful in the timely identification of pulmonary complication and for practical troubleshooting at the cot-side.

## Introduction

Tidal capnography is a non-invasive method to continuously measure exhaled carbon dioxide (CO_2_) via a CO_2_ sensor which is placed between the endotracheal tube (ETT) and the ventilator circuit. Historically, there has been some scepticism on the accuracy of capnography in neonatal care, based on technological limitations rising from the very small tidal volumes and high breathing rates of neonates, especially the more preterm ones. Recent advances have largely addressed these limitations and have enabled more widespread use of capnography within neonatal care (1).

Maintenance of CO_2_ within the desired range is clinically important in the newborn to avoid the adverse outcomes associated with too high or too low CO_2_ levels (2, 3). As CO_2_ is a potent moderator of cerebral vascular tone, both extremes can lead to detrimental side effects such as intraventricular haemorrhage (IVH) and periventricular leucomalacia (4). Moreover, fluctuations of CO_2_ during the early days of life or during initial stabilisation in the delivery room have been strongly associated with the development of IVH (5).

Waveform capnography can provide information over and above a solitary value of exhaled carbon dioxide, and it is important that clinicians at the bedside can interpret the contemporaneous and stored/trend information which can be provided by the capnography waveform (6). Clinical real-time interpretation of waveforms may identify life threatening complications of mechanical ventilation such as dislodged or blocked ETT (7), or describe established or impending lung pathologies (8). The CO_2_ waveforms are more useful when evaluated with the concurrent flow, volume and pressure waveforms (9), although not all ventilators present them at a synchronous scale. This short overview aims to cover the practical basics of neonatal capnography, providing an aid for clinicians asking the following question: “*In newborn infants requiring mechanical ventilation, what value does the capnography waveform have in supporting diagnosis and clinical troubleshooting?”*

### Mainstream vs. sidestream

Both main- and side- stream capnographs are placed in-line with the ventilator circuit, between the ETT and ventilator tubing (10). The difference between them is where the CO_2_ is analysed. In mainstream capnographs, there is an infra-red sensor that sits in-line with the capnograph, and analysis is undertaken in real time. In sidestream capnographs, there is a sample line which exports exhaled gas to an infra-red analyser away from the ventilator circuit (8). There can be a small time delay in reading due to the transit time of the CO_2_ molecules along the sample line to the infra-red sensor. Importantly, with the sidestream capnograph the waveform may be affected by water droplets within the sample line (11), which form as the side-stream tube is cooled outside the incubator, especially in extremely preterm infants who require high incubator humidity.

### Phases of the capnogram

**Figure 1:**
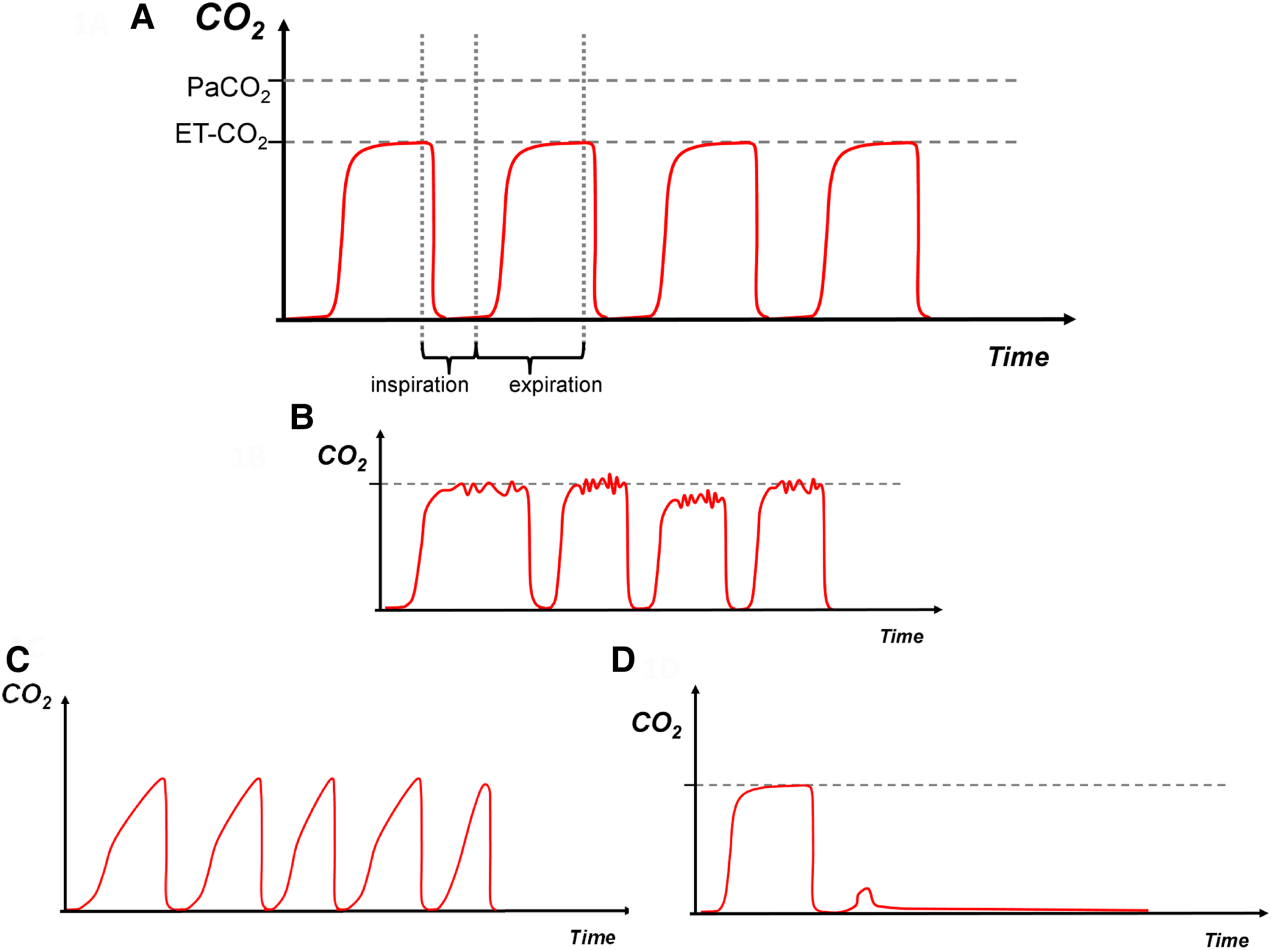
(**A**)—normal, (**B**)—water in the circuit, (**C**)—increased expiratory resistance, (**D**)—dislodged endotracheal tube. Carbon dioxide on *y*-axis with time on *x*-axis. The red trace depicts the capnography waveform. PaCO_2_: arterial CO_2_, EtCO_2_: end tidal CO_2_.

The capnogram is divided into three phases (Figure 1A). Phase I is the first part of expiration during which gas is expired from the large conducting airways, this phase contains little or no carbon dioxide. Phase II is the second part where there is mixing of gas from the large and small airways, thus exhaled carbon dioxide levels rapidly rise. Phase III is the final part which contains alveolar gas; it is this phase where the end-tidal carbon dioxide (EtCO_2_) value is determined. A steep phase III occurs if there is ventilation inhomogeneity, as different lung units with differing time constants empty unevenly during expiration (12). The alveolar plateau might be shorter in preterm infants with high breathing rates or absent in spontaneous breaths during synchronised ventilation as expiration might finish before complete emptying of the alveolar gas: in these cases, the EtCO_2_ will underestimate the “true” alveolar CO_2_. Interpretation of neonatal tidal capnography should also take into account that in ventilated preterm infants, the correlation between arterial and EtCO_2_ is acceptable but the agreement is suboptimal with a fixed mean difference of 0.54 kPa between arterial and EtCO_2_ values (13). Of note, this correlation is negatively influenced by the severity of lung disease, with lower correlation in more severe lung disease (13). Although rarely used in neonatal clinical practice, exhaled CO_2_ can also be plotted against the expired tidal volume (volumetric capnography) and the resulting capnograms can be used to calculate respiratory dead-space and quantify ventilation inhomogeneity (14).

## Scenarios

We present some graphic representations of commonly seen time-based capnograms, based on observed patterns.

### Normal

Conventional mechanical ventilation inflations in a term infant without respiratory pathology. Inspiratory baseline during phase one followed by a sharp vertical upstroke during the initial expiration of gas from the large conducting airways, followed by a relatively horizontal plateau phase. It is expected that the EtCO_2_ is lower than the PaCO_2_ due to some degree of respiratory dead space (Figure 1A).

### Water in the circuit

Water droplets or humidity within the ventilator tubing can lead to turbulent flow and a non-smooth trace as interference occurs (Figure 1B). Removal of the droplets will usually smoothen the signal.

### Increased expiratory resistance

There is a consistent upward slant in the third phase, which can be referred to as a “shark fin” appearance. Commonly seen in cases of ventilation inhomogeneity and increased expiratory resistance such as in bronchopulmonary dysplasia (Figure 1C).

### Dislodged endotracheal tube

Sudden drop of the waveform trace with loss of EtCO_2_ as no expired CO_2_ is detected, may occur if the ETT is dislodged, blocked kinked, or when the ventilator is disconnected from the infant (Figure 1D). This pattern can also occur if there is sudden blockage of the ETT or the infant splints its respiratory muscles enough to prevent an inflation.

### Rebreathing

**Figure 2:**
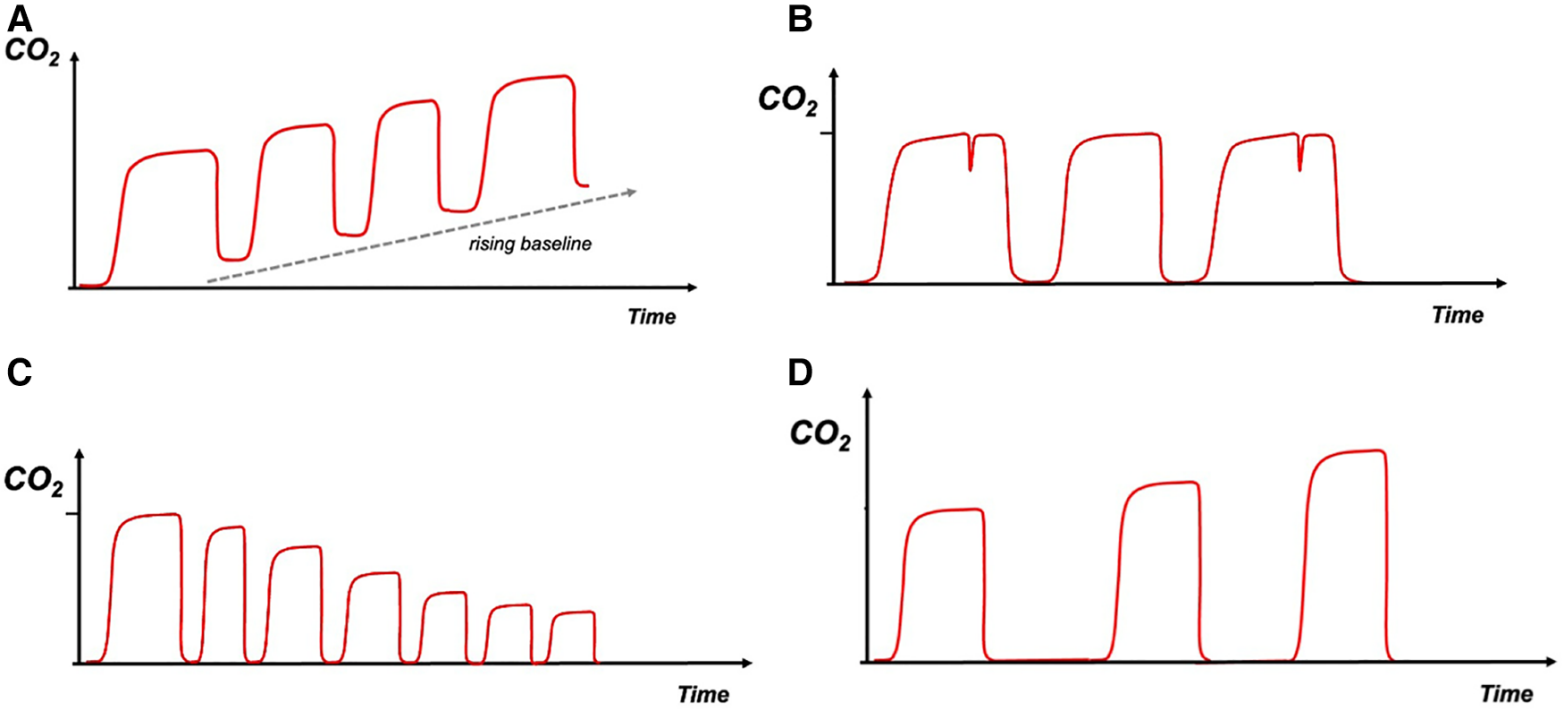
(**A**)—rebreathing, (**B**)—infant asynchrony, (**C**)—hyperventilation, (**D**)—hypoventilation. Carbon dioxide on *y*-axis with time on *x*-axis. The red trace depicts the capnography waveform.

Rising baseline with EtCO_2_ not returning to zero during inspiration; increased re-breathing of CO_2_ (breathing of expired gas) occurs from low tidal volume or increased apparatus dead space, leading to ineffective removal of CO_2_ from the circuit. The EtCO_2_ value rises with consecutive breathing cycles (Figure 2A).

### Infant asynchrony

Breathing out of synchrony with the ventilator, could cause a “beaking” pattern on the waveform as the infant tries to inspire during expiration. This pattern can also be seen in cases of inadequate flow to trigger the ventilator (Figure 2B). This type of asynchrony could also be evaluated by the flow and volume waveforms.

### Hyperventilation

Decreasing EtCO_2_ level and speeding up of the breathing frequency are observed (Figure 2C).

### Hypoventilation

Increasing EtCO_2_ level and slowing down of the breathing frequency are observed (Figure 2D).

## Discussion

We have presented a practical approach to neonatal tidal capnography used as a clinical adjunct to respiratory monitoring at the cot-side. With the emergence of continuous tidal capnography as a reliable monitoring tool in neonatal intensive care, we believe that the general neonatal audience would benefit from this targeted education on the interpretation of CO_2_ waveforms.

It is interesting to note that similarly to the clinical estimation of the work of breathing and the utilisation of blood gases and chest radiographs, waveform monitoring holds unique information that describes the respiratory condition of a single subject at a specific time point (1). For example, the slope of phase III of a time capnogram, holds information on ventilation inhomogeneity which is not available from the waveforms of flow, volume or pressure, chest radiograms or blood gas analysis. Although interpretation of the CO_2_ waveforms can be undertaken taking into consideration the concurrent waveforms of pressure, flow and volume (7), this approach is not always possible in everyday clinical practice as some ventilators do not display the CO_2_ traces in synchrony with volume and flow, while some other units display the CO_2_ traces in the monitoring screen rather that the ventilator display.

We should note that unlike some other methods of respiratory monitoring, tidal capnography is non-invasive and provides real-time breath by breath information.

Since CO_2_ fluctuations during initial stabilisation in the labour ward have been associated with IVH (5), there is significant potential in using capnography as part of standard monitoring after birth. In the current era, furthermore, the majority of preterm infants are managed mostly on non-invasive respiratory support, and during this period high levels of CO_2_ can be implicated in impaired oxygenation (15). There is scope, thus, in expanding the use of CO_2_ monitoring during non-invasive support, which will require the future development of novel interfaces and lighter capnographs.

Tidal capnography is arguably not the only way to monitor CO_2_ and is not possible in high frequency modes due to very high breathing rates. An alternative monitoring method is via transcutaneous CO_2_ measurement, which measures CO_2_ at the tissue level and the value is thus closer to the arterial one (16). Tidal CO_2_ measurements can be influenced by the settings and mode of ventilation as a high breathing frequency may exceed the capnograph's response capabilities, while a significant ETT leak, high airway resistance and respiratory rate may also decrease the accuracy of the measurement (17).

In conclusion, we have described patterns of clinical scenarios where tidal capnography can be useful in diagnosis and troubleshooting at the cot-side.

## Data Availability

The data for this study are available for the corresponding authors upon reasonable request.
